# Impact of Crosslinking on the Characteristics of Pectin Monolith Cryogels

**DOI:** 10.3390/polym14235252

**Published:** 2022-12-01

**Authors:** Aleksandra Nesic, Sladjana Meseldzija, Antonije Onjia, Gustavo Cabrera-Barjas

**Affiliations:** 1 Department of Chemical Dynamics and Permanent Education, Vinca Institute of Nuclear Sciences—National Institute of the Republic of Serbia, University of Belgrade, Mike Petrovica-Alasa 12-14, 11000 Belgrade, Serbia; 2 Department of Analytical Chemistry and Quality Control, Faculty of Technology and Metallurgy, University of Belgrade, 11000 Belgrade, Serbia; 3 Unidad de Desarrollo Tecnológico (UDT), Universidad de Concepción, Avda. Cordillera 2634, Parque Industrial Coronel, Coronel 4190000, Chile

**Keywords:** pectin, cryogels, alcohol gelation, biodegradation

## Abstract

In this research, the pectin monoliths were prepared via the sol-gel process through different routes of crosslinking and additional freeze-drying. The crosslinking reaction was induced by the use of calcium ions in aqueous solutions and in alcohol/water solutions. The resulting pectin monoliths obtained by freeze-drying were macroporous with open cells, limited specific surface area, moderate mechanical stability and moderate biodegradation rate. The presence of alcohol in crosslinking solution significantly changed the morphology of final pectin monoliths, which was evidenced by the reduction of their pore size for one order. The specific surface area of pectin monoliths obtained through the calcium-water-alcohol route was 25.7 m^2^/g, the Young compressive modulus was 0.52 MPa, and the biodegradation rate was 45% after 30 days of immersion in compost media. Considering that pectin can be obtained from food waste, and its physical properties could be tailored by different crosslinking routes, the pectin monoliths could find wide application in the pharmaceutical, agricultural, medical and food industries, providing sustainable development concepts.

## 1. Introduction

Pectin belongs to the group of heteropolysaccharides and is one of the most abundant cellular components of the plant wall. It consists of homogalacturonans (HG), xylogalacturonans (XGA), rhamnogalacturonans type I (RG-I), rhamnogalacturonans type II (RG-II), arabinans and arabinogalactans [1]. The largest part of the pectin backbone (60–70%) consists of homogalacturonan, i.e., of homopolymers of D-galacturonic acid, connected by α (1→4) glycosidic bonds, where some of the carboxyl groups are methyl-esterified, while some O–2 and O–3 positions might be O–acetylated [2]. Pectin can be found in the pulp and peel of lemons, oranges, limes, sunflowers, apples, potatoes, tomatoes, and carrots. Commercially, pectin is mostly obtained from citrus fruits, as well as from apple pomace, as secondary products in the production of juices [3]. Depending on the source, pectins can differ in molecular weight, degree of acetylation and methylation, as well as in the content of galacturonic acid and neutral sugars. One of the unique properties of pectin is that it can be physically and/or ionically crosslinked by external stimuli, thus easily forming a three-dimensional hydrogel network. The degree of esterification affects pectin gelation and processing conditions, so pectin with a high degree of methyl-esterification is sensitive in an acidic environment and gels at low pH values and in the presence of high concentrations of sugars. On the other side, pectin, with a low degree of methyl-esterification, has the ability to form gels in the presence of divalent cations, and the crosslinking reaction is described by the egg-box model [4]. Moreover, in both cases (high-methylated and low-methylated pectin), it is confirmed that higher temperature improves the gelation ability. Hence, hydrogen bonds, as well as hydrophobic interactions, have an important role in the gelation of pectin [5].

The ability of pectin to undergo gelation by different external stimuli using sol-gel technology allows the production of three-dimensional networks in the form of hydrogels (wet gels), xerogels (vacuum drying), aerogels (supercritical CO_2_ drying) and cryogels (freeze-drying). The main characteristic of wet gels is their ability to bind substantial amounts of water, thus increasing their volume. The drying route of wet gels dictates the textural (porosity, pore size distribution) and functional properties of final porous networks and their application potential. During the drying process under vacuum comes pore collapse, so final materials usually possess high density and low porosity [6]. On the other side, it has been demonstrated that supercritical drying preserves the morphology of wet gels, giving mesoporous materials with low density [6,7,8]. Furthermore, by freeze-drying, it is possible to tailor the macropore and mesopore space within the final material [9]. These properties, along with the non-toxicity, biodegradability and biocompatibility, make pectin-based materials adequate for a wide range of applications such as agriculture, pharmacy, food and biomedicine. Up to date, pectin-based three-dimensional networks have been investigated for tissue engineering [10], drug delivery [11], wound healing [12,13], food technology [14] and as food packaging material [15].

The aim of this work is to study the influence of different crosslinking routes of pectin on the physical properties of final materials. Namely, three different crosslinker solutions were used: (a) aqueous calcium bath, (b) tert-butanol bath, and (c) calcium-tert butanol/water bath. The obtained hydrogels were freeze-dried, and their textural, morphological, mechanical, and thermal properties were evaluated. It was previously demonstrated that pectin and alginate could gel in the presence of different alcohols, such as ethanol, methanol, propanol and 1-butanol and that obtained hydrogels can easily be turned into mesoporous aerogels by supercritical drying [16,17,18]. In this work, tert-butanol was chosen as a precursor for gelation since it is widely used as a green solvent and is proven as an efficient crosslinker for various polysaccharides, providing the cryogels with different levels of hierarchy, i.e., tailoring the macro and mesopores in the system by different used concentrations [9]. Up to date, there are several papers reported in the literature that deal with the characterization of pectin-calcium crosslinked aerogels or cryogels [6,7,8,19,20], but to the best of our knowledge, there is no published data related to the influence of tert-butanol and calcium ions combined together on physical properties of final materials. Moreover, there is no comparison of the impact of different crosslinking routes (including ionic and alcohol-induced crosslinking, separately and combined) on the final properties of the pectin of the same origin. Finally, since pectin is a natural polymer derived from renewable resources, the aerobic degradation rate of all obtained pectin-based cryogels was assessed in order to evaluate if these materials can contribute to the sustainable development goals.

## 2. Materials and Methods

### 2.1. Chemicals

Citrus pectin, with a degree of methylation of 50%, was obtained from Herbstreith & Fox KG, Pektin-Fabriken (Neuenburg, Germany). Tert-butanol and CaCl_2_ × 2H_2_O were purchased from Sigma Aldrich (St. Louis, MO, USA).

### 2.2. Preparation of Cryogels

Pectin-based cryogel monoliths were prepared through three different crosslinking routes in a two-step process. In all cases, the primary step was the dissolution of pectin in an aqueous solution (2.0 wt%) at room temperature. The first gelation route was by use of CaCl_2_ aqueous solution (P1 sample), the second gelation route was by use of tert-butanol (P2 sample), and the third gelation route by use of tert-butanol/water solution containing CaCl_2_ (P3 sample). The final concentration of calcium ions in samples P1 and P3 was 2 wt%, with respect to the mass of pectin, whereas the final concentration of tert-butanol in sample P3 was 40 wt%. The crosslinking reaction occurs through slow diffusion of crosslinking solution into pectin solution. The mixture was left until complete gelation, which took place after 4 h. The obtained pectin gel samples were frozen in liquid nitrogen and freeze-dried for at least 24 h on a VirTis SP Scientific Sentry 2.0 freeze drier (New Life Scientific Inc, Woonsocket, RI, USA). The drying conditions were as follows: vacuum set to 100 m Torr and a condenser temperature of −80.0 °C.

### 2.3. Morphology and Texture of Cryogels

The morphology of the prepared samples was analyzed by FEI Quanta 200 FEG Scanning Electron Microscope (SEM) (FEI Company, Hillsboro, OR, USA) at an accelerating voltage of 5–10 kV. Prior to the SEM analysis, the cryogels were sputtered with gold (~7 nm). The bulk density of the samples was calculated as the mass-to-volume ratio, and the presented results are an average of three measurements. The specific surface area was analyzed by nitrogen adsorption-desorption test (Nova 3000e surface area analyzer, Quantachrome Instruments, Bointon Beach, FL, USA) and mercury porosimetry test (Carlo Erba Porosimeter 2000 equipped with the software Milestone 200, Carlo Erba, Washington, DC, USA). Before the measurements, the samples were degassed at 75 °C for 24 h. The presented results are the average values of 3 measurements; the standard deviation was within 10%.

### 2.4. Calcium Content in Cryogels

An accurately weighed amount of cryogel was digested with hydrogen peroxide (30%, *w*/*w*) and nitric acid (70%, *w*/*w*) in a microwave oven (working conditions: power = 800 W; temperature = 200 °C, ramp 10 min, working time = 20 min). Upon cooling, the solution was diluted up to 50 mL in a volumetric flask using deionized water. Appropriate dilutions were carried out, and the calcium ion content in the cryogel was determined by the use of Inductively coupled plasma mass spectrometry (ICP–MS) with a quadrupole detector at ^44^Ca. The presented results are the average values of 3 measurements; the standard deviation was within 5%.

### 2.5. Water Uptake and Solubility of Cryogels

The water uptake (WU, %) of cryogels was determined by the gravimetric method. Firstly, the cryogels were weighed (m_0_, g) and placed in 100 mL of distilled water at room temperature (25 °C). The cryogels were taken out from the water after 24 h; the excess water from the surface was removed by filter paper, and the weight of the swollen cryogels (m_t1_, g) was measured. The water uptake was calculated by the following equation:

(1) $$\mathrm{WU}\;\left(\%\right)=\frac{\left(m_{t1}-m_{0}\right)\times 100}{m_{0}}$$

The solubility test was determined as the content of dry matter solubilized after 24 h in distilled water. The swollen cryogels were taken out after 24 h and dried until constant weight (m_t2_, g) in an oven at 105 °C. The solubility degree (SLD) was calculated according to the following equation:

(2) $$\mathrm{SLD}\left(\%\right)=\frac{\left(m_{0}-m_{t2}\right)}{m_{0}}\times 100$$

The presented results are the average values of 3 measurements; the standard deviation was within 10%.

### 2.6. Mechanical Analysis

The uniaxial compression test was performed according to the ASTM D695 standard. Cylindrical specimens with a height-to-diameter ratio of approximately 5 were used. A load of 10 kN was applied. The samples were compressed upon the breaking point at a compression rate of 2 mm/min. The force-time curves were converted to the compressive stress (σ_c_, MPa)-compressive strain (ε_c_, %) curves and Young’s modulus (E_c_, MPa) was obtained from the linear part of the curve. The results are presented as the average of five experiments, and values of compressive strength and Young’s modulus are within ±15%.

### 2.7. Thermal Analysis

Thermogravimetric analysis (TG) was performed using a TGA Q500 (TA Instruments New Castle, DE, USA) instrument under dynamic nitrogen flow in the temperature range from 25 to 600 °C. The nitrogen flow rate was 60 cm^3^/min while the heating rate was 10 °C/min. The weight of the samples was approximately 10 mg.

### 2.8. Biodegradation

The aerobic biodegradation of pectin monolith cryogels was determined according to the ISO 14855-2:2018 standard. Each sample (~0.2 g) was ground, mixed with compost soil and stored in a 250 mL sample chamber at room temperature. The amount of released CO_2_ within the time was monitored and measured by Micro-Oxymax Respirometer (Columbus Instruments, Columbus, OH, USA). The degree of biodegradation was calculated according to the following equation:

(3) $$D\;=\frac{V_{1}-V_{b}}{V_{2}}\times 100$$

where V_1_ and V_b_ are the amount of CO_2_ released within the sample reactor and the blank reactor, respectively, and V_2_ is the theoretical amount of CO_2_ available from the samples. The presented results are the average values of 3 measurements; the standard deviation was within 10%.

## 3. Results

### 3.1. Morphology and Texture of Cryogels

**Figure 1:**
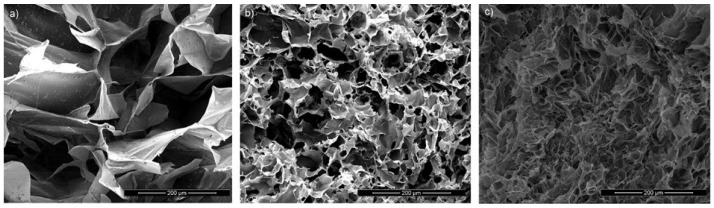
SEM morphology of cryogels: (**a**) P1, (**b**) P2 and (**c**) P3.

Pectin monolith cryogels were obtained by gelation in (a) an aqueous solution of calcium ions (2 wt%), (b) tert-butanol, and (c) a solution that combines calcium ions and tert-butanol/water solution. The morphology of obtained cryogels is presented in Figure 1. The pore size distribution of all samples was evaluated by scanning 3 pieces of each sample at different magnifications and presented in Table 1. It can be seen that all pectin monolith samples have dense sheet-like morphology with some level of interconnected voids. This type of structure is common when the slow growth of crystals occurs, caused by slow freezing and slow drying process. A similar structure is reported for other pectin cryogel systems in literature [6,19] and for other biopolymer-based cryogels, such as alginate [21,22] and starch [23,24]. It is interesting to note that the structure is dense in samples that contain tert-butanol (P2 and P3). Namely, the range of diameter of voids for samples crosslinked only in calcium ion solution (P1) is between 150 and 400 µm, whereas in the case of samples crosslinked in tert-butanol (P2) and Ca/tert-butanol-water (P3), the range is between 10 and 50 µm. Furthermore, the bulk density of the P2 and P3 (0.1–0.12 g cm^−3^) samples is higher than for sample P1 (0.06 g cm^−3^). It appears that the presence of tert-butanol in crosslinking solution increases the hydrophobic interactions of pectin chains and leads to interpolymer bridging, thus changing the morphology and texture of final cryogels.

In order to get an idea about the specific surface area of samples, a mercury porosimetry test and nitrogen adsorption test was performed, and these results are presented in Table 1. Mercury porosimetry is a generally used method to characterize the texture of materials with large macropores. The pressure of mercury causes the contraction of biobased cryogels, which can cause the destruction of pores, so valuable information can be obtained sometimes only at smaller applied pressures [25]. However, during the measurement, not only the compression of samples occurs but also the partial crushing of cryogels, so the pore size distribution can not be determined by this technique. On the other side, the nitrogen adsorption test is not precise enough for the evaluation of pore volume and pore size distribution of materials that are macroporous. Hence, both techniques can give just an idea about the specific surface area of obtained samples and its relationship between the samples, i.e., similarities or differences among the samples.

It is demonstrated that gelation in the presence of tert-butanol (sample P2 and P3) provides materials with higher surface areas in comparison to the pectin crosslinked in an aqueous solution of calcium ions. The obtained specific surface area of pectin cryogels is slightly higher by Hg porosimetry than by nitrogen adsorption test, and it ranges between 17 and 26 m^2^ g^−1^. These values are higher than for the starch freeze-dried cryogels (7.7 m^2^ g^−1^) [23] and have a similar range for the other reported pectin cryogel systems (10–20 m^2^ g^−1^) [6]. Apparently, the specific surface area originates from the porosity in the walls of voids. Similar results are obtained in literature for the starch cryogels and resorcinol-formaldehyde cryogels, where measurable, specific surface area is obtained by nitrogen adsorption test, but SEM micrographs displayed macroporous systems with large macropores of the 200 µm [23,26]. According to the SEM and Hg porosimetry/nitrogen adsorption test results, the highest specific surface area and lowest pore size distribution, but higher density exhibits the pectin-based cryogel crosslinked by the solution of Ca/tert-butanol-water. The presence of alcohol induces the re-organization of macromolecular chains, with competitive interactions of; (a) self-association, i.e., interpolymer bridging through intermolecular hydrogen bonding and hydrophobic interactions, and (b) ionic crosslinking with the Ca-ions. It has been demonstrated before that the crosslinking of alginate in the alcoholic bath (methanol) forms the association structures through interpolymer bridging [18]. Moreover, Rodriguez-Dorado et al. have reported that alginate aerogels crosslinked in Calcium-ethanolic solution exhibit higher specific surface area than alginate microbead aerogels crosslinked in Calcium-aqueous solution [27].

### 3.2. Water Uptake, Solubility and Calcium Content in Cryogels

The water uptake and solubility of pectin-based monolith cryogels after 24 h of immersion in distilled water, as well as the content of calcium ions in them, are presented in Table 2. As it is shown, the pectin sample crosslinked only by alcohol dissolves after 24 h, confirming that occurred crosslinking reaction is only physical, through intermolecular hydrogen bonding, thus making these cryogels less stable in aqueous solutions, in comparison to the ionically crosslinked cryogels. On the other side, the solubility of the P1 and P3 samples is only 5% after 24 h of immersion in distilled water. The water uptake for these two samples is in the range between 346 and 983%, proving that these samples have the ability for large water uptake, which makes them suitable for the range of applications in the food, agriculture and biomedical sector. It is interesting to note that water uptake is significantly higher for the P1 sample than the P3 sample. The presence of tert-butanol and Ca in cryogel induces lower uptake of water due to the formation of a denser network. As it is confirmed by SEM, in the presence of Ca/tert-butanol-water crosslinker, it comes to more dense morphology of pectin cryogels, with less interconnected voids, when compared to only Ca-crosslinker. In addition, this result can be related to calcium ion content entrapped into pectin cryogel. Namely, it is shown that the P3 sample contains more calcium ions than the P1 sample. Apparently, the competitive interaction of tert-butanol and Ca-ions with pectin chains induces the better physical re-organization of chains, allowing better entrapment of calcium ions in comparison to the samples crosslinked only by calcium ions.

The main ionic crosslinking process of pectin is described by the physical entrapment of divalent cations (in this case, calcium ions) between non-methyl esterified galacturonate units from pectin, where the junction zones are formed in so-called “egg box” model [28]. Further research has confirmed that ionic crosslinking does not involve only physical entrapment but also the interaction of calcium ions with the oxygen atom from the carboxylate group of pectin, making the stable three-dimensional and thermos-irreversible network [29]. It has been also demonstrated that, at some level, hydrophobic interactions also contribute to network development [30,31]. On the other side, Oakenfull et al. have shown that alcohol presence can stabilize the junction zones to form three-dimensional pectin networks through hydrogen bonds and hydrophobic interactions of ester methyl groups from pectin [5]. In both crosslinking processes, the stabilization of hydrogel, i.e., the gelation process, depends on the degree of methylation and distribution pattern of these groups. Taking into consideration the results presented in Section 3.1 and in Table 2, it can be concluded that the presence of tert-butanol and calcium ions in crosslinking bath has a synergic effect on developing a more stable three-dimensional network.

### 3.3. Mechanical Properties

**Figure 2:**
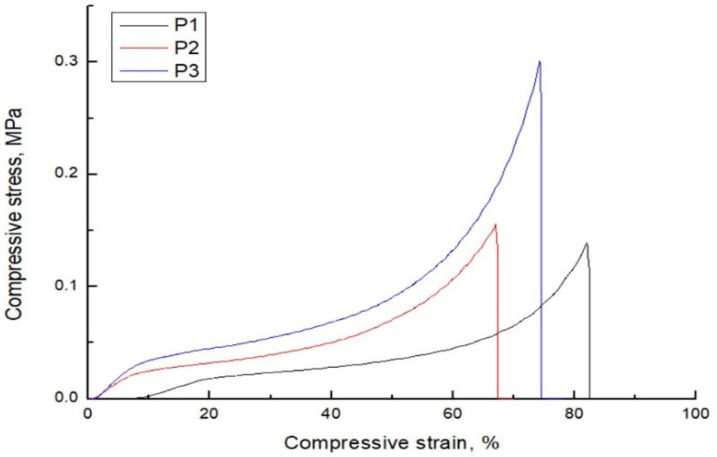
Compressive stress–strain behavior for pectin-based cryogels.

Mechanical properties of pectin-based cryogels, in terms of compressive Young Modulus and compressive strength, were studied by uniaxial compression test and presented in Figure 2. The Young compressive modulus represents the level of stiffness or resistance to deforming until applied load (compression). In this work, the obtained Young modulus values for samples P1, P2 and P3 are 0.18 MPa, 0.33 MPa and 0.52 MPa, respectively. This trend is predictable due to pore size distribution and density results obtained for pectin-based cryogels (see Section 3.1). Generally, the materials with higher porosity and lower density negatively impact the Young modulus in comparison to the materials with a more dense structure and less porosity. Moreover, it is demonstrated in the literature that ethanol, used as a co-solvent in calcium crosslinking solution, improves the mechanical strength of alginate films in comparison to the film crosslinked with an aqueous solution of calcium ions [32]. The maximum compressive strength represents the maximum compressive stress that the material can withstand until it breaks. The lowest compressive strength (0.14 MPa) is obtained for the P1 sample, whereas the highest compressive strength exhibits the sample P3 (0.3 MPa). Also, it is important to note that all samples plastically deform until strain between 67% and 82%. Overall, the presence of tert-butanol and calcium ions presents a synergic effect for the formation of more mechanically stable three-dimensional networks. Chen et al. have reported the Young compressive modulus of neat pectin cryogels between 0.04 (2.5 wt% content of pectin in cryogel) and 48 MPa (15 wt% content of pectin in cryogel) [19]. Moreover, the addition of clay significantly improved the mechanical performances and Young modulus of pectin cryogels, reaching a range between 0.16 and 114 MPa. Furthermore, Yang et al. have obtained a Young modulus of neat pectin cryogels of 0.09 MPa, and enhanced by 119% with the inclusion of boron nitride nanosheets into the pectin matrix [33]. The difference in Young modulus value presented in this work and literature is due to different types of used pectin and different degree of methylation of pectin. Namely, it is proved that degree of methylation, but also the distribution pattern of methylated and non-methylated galacturonic units, influences the pectin hydrogel strength [4,34,35,36]. In addition, the pectin concentration used to make hydrogels and cryogels give contribution too to the mechanical strength of final materials. In this work, the cryogels are made of 2 wt% of pectin because the focus was to evaluate the lowest possible concentration of raw material. i.e., pectin to obtain cryogels without any visible surface damage and to characterize them. Our preliminary results (not presented here) showed that cryogels with pectin content of 1 wt% and 1.5 wt% had visible cracks and “peeling off” surfaces. On the other side, cryogels reported in literature usually contained 2.5–6 wt% of pectin, which provided better mechanical stability. Nevertheless, the influence of different pectin concentrations on the physical-chemical properties of cryogels obtained by the most efficient crosslinking route established in this work will be assessed in the future and part of the forthcoming paper.

### 3.4. Thermal Analysis

**Figure 3:**
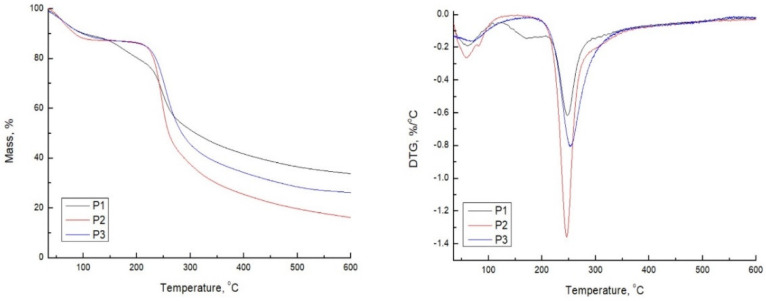
TGA/DTG of pectin-based cryogels.

There are three types of absorbed water in hydrophilic materials: free unbound water, freezing bound and non-freezing water or bound water. Free water does not interact, via hydrogen bonding, with the polymeric chains, and it is released from samples up to 100 °C. The freezing-bound water interacts only weakly with the polymeric chain, while the non-freezing water is represented by molecules of water bound to the polymeric chains through hydrogen bonds [37]. Generally, these two types of water are released from samples between 100 °C and 200 °C. On the basis of the above claims, it can be concluded by the analysis of the pectin-based cryogels thermograms (Figure 3 and Table 3) that the mass loss in the first region up to 100 °C is associated with the evaporation of free water (or free absorbed moisture), while the second region up to 200 °C represents a loss of freezing and non-freezing bound water. All tested samples contain free water because they are macroporous materials subjected to the fast absorption of moisture. The highest content of bound water is in sample P1, according to the weight loss percentage at 200 °C (see Table 3, W_L200_, %). This result is expected since it is already confirmed by SEM that pectin crosslinked only by calcium ions provides the materials with the highest pore size distribution and less dense structure, thus being more subjected to moisture absorption in comparison to the samples that contain tert-butanol. In all cases, the decomposition process of pectin chains starts above 200 °C, which includes the random split of the glycosidic bonds, vaporization and elimination of volatile products [1,15]. It is interesting to note that there is a significant shift of Tonset (the onset temperature at which starts degradation) to lower values for samples P2 and P3 when compared to the P1 sample, indicating that hydrogen bonds formed between pectin chains are easier to break at increasing temperature, than bonds formed ionically with calcium ions. However, it is important to highlight that the second and third step of degradation of the P1 sample is a continuous process, where it is not possible precisely to say where one degradation step ends, and another starts. In the case of samples P2 and P3, the onset temperature of the third degradation step is clear since these two samples do not contain too much-bound freezing and non-freezing water; hence no continual steps are occurring. Although the main degradation of pectin chains starts earlier for cryogels that contain tert-butanol, the Tdeg is shifted to a higher value for the P3 sample and to a lower value for the P2 sample, in comparison to the P1. This result confirms once again that the self-associated hydrogen bonds of pectin chains are less thermally stable than bonds occurred by ionic crosslinking of calcium ions with pectin chains and bonds formed in combination with ionic (Ca^2+^) and physical (tert-butanol).

### 3.5. Biodegradation

**Figure 4:**
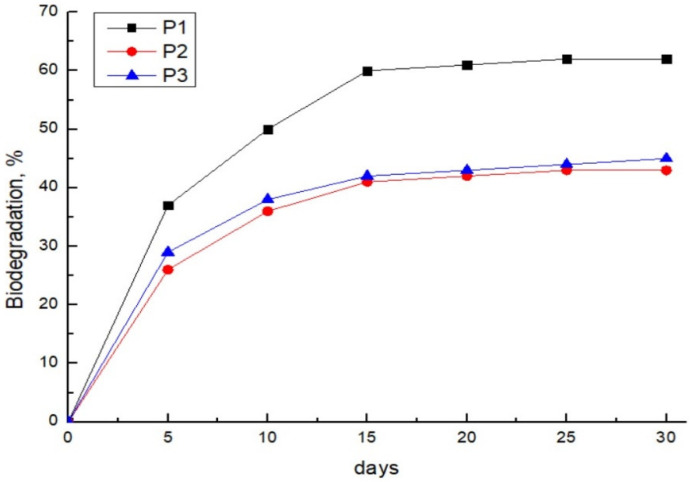
Aerobic biodegradation of pectin-based cryogels.

Biodegradation of materials is essential for the balance of nature. Although there is increased awareness about the sustainable development of biodegradable materials and promotion of new approaches to replace plastic materials or upgrade them to be biodegradable, there are still not enough data in the literature related to this topic, where is studied on which conditions polysaccharides degrade, and how to speed their process of degradation. Hence, in this work, the aerobic biodegradation of pectin-based cryogels was performed by a respiratory method and monitored within the period of 30 days, and results are presented in Figure 4. As can be seen, the aerobic biodegradation rate is in the range of 43 and 62%, and the equilibrium plateau is reached within 15 days. The highest rate of aerobic biodegradation has sample P1, whereas there is no significant difference in the biodegradation rate between samples P2 and P3. It appears that the presence of tert-butanol decreases the aerobic biodegradation of pectin cryogels. This result is expected since it is well known that samples with higher moisture/water content promote the propagation of microorganisms, which as a result, increase the respiration rate, as well as biodegradation. A similar biodegradation rate determined by the respiratory method is obtained for the polysaccharide cryogels. Chen et al. have demonstrated that control pectin cryogel reaches a biodegradation rate of 60%. However, the equilibrium plateau is reached within 10 days, which is faster than in this work. Also, Praglowska et al. obtained a biodegradation rate of 80% for chitosan cryogels within 11 days of immersion in compost media [38]. On the other side, the alginate-calcium xerogel beads degrade only 32% after 2 months [39], ammonium alginate-phytic acid cryogel 92% after 45 days [40], cellulose powder degrades 83% after 45 days [41].

## 4. Conclusions

In this work, calcium ions and tert-butanol, separately and in combination, were used as the precursors for crosslinking of pectin. The hydrogels were freeze-dried and subjected to several different characterization techniques in order to evaluate their textural, morphological, mechanical, thermal and biodegradable property. It was shown that a combination of tert-butanol and calcium ions provides materials with higher density but also higher specific surface area and smaller pore size distribution. The highest Young modulus was obtained for this sample, too and reached a value of 0.55 MPa. On the other side, tert-butanol had a negative influence on the thermal stability of pectin-based cryogels, causing the shift of the onset degradation temperature to lower values of 7 °C. Moreover, the biodegradation rate of pectin-based samples that contain tert-butanol was 45% after 30 days of immersion in compost media, whereas the pectin sample crosslinked by calcium ions had a biodegradation rate of 62%. The results obtained in this work demonstrate that different crosslinking routes can significantly impact the morphology and also final properties of materials, dictating the direction of application. Pectin, as a natural polymer from renewable sources, presents a suitable feedstock for the processing of sustainable and biodegradable materials with tailored macroporosity through different crosslinking routes.

## Figures and Tables

**Table 1:** Characterization of pectin-based cryogels by nitrogen adsorption.

Sample	Specific Surface Area, m^2^ g^−1^		Average Pore Size, µm	Density, g cm^−3^
	Hg Porosity	BET	SEM	
P1	17	8	150–400	0.06
P2	23	14	10–50	0.10
P3	26	19	10–50	0.12

**Table 2:** Water-related properties of pectin-based cryogels.

Sample	WU, %	SLD, %	Ca Content, ppm
P1	983	5	70
P2	dissolved	100	–
P3	346	5	103

**Table 3:** Thermal properties of pectin monolith cryogels, where W_L100_ and W_L180_ present the weight loss at 100 °C and 200 °C, respectively, Tonset is the temperature at which starts degradation, and Tdeg is the temperature of maximum degradation rate.

Sample	W_L100_, %	W_L200_, %	Tonset, °C	Tdeg, °C	Char Residue at 600 °C, %
P1	10	20	216	248	34
P2	12	13	212	245	16
P3	11	14	209	252	26

## Data Availability

Not applicable.
